# Pseudo-Thrombotic Microangiopathy Secondary to Vitamin B12 Deficiency

**DOI:** 10.7759/cureus.73620

**Published:** 2024-11-13

**Authors:** Joana Tavares Pereira, João Barbosa Barroso, Ana Azevedo, Andreia Ribeiro, Carla Tonel

**Affiliations:** 1 Internal Medicine, Hospital Vila Franca de Xira, Lisboa, PRT; 2 Nephrology, Hospital Vila Franca de Xira, Lisboa, PRT

**Keywords:** cobalamin deficiency, pancytopenia, pernicious anemia, thrombotic microangiopathies, vitamin b12 deficiency

## Abstract

Vitamin B12 deficiency can present with hematological and neuropsychiatric manifestations and is most commonly due to pernicious anemia in adults. A rare presentation is pseudo-thrombotic microangiopathy, which mimics thrombotic microangiopathies and may result in unwarranted treatment, including plasmapheresis.

This condition must be considered in patients presenting with features of microangiopathic hemolytic anemia, and prompt treatment with vitamin B12 supplementation should be initiated. Although the laboratory findings of pseudo-thrombotic microangiopathy are similar to those of thrombotic thrombocytopenic purpura, there are some significant differences, such as in the reticulocyte production index.

This report presents the case of a patient with pseudo-thrombotic microangiopathy caused by vitamin B12​​​​​​​ deficiency.

## Introduction

Vitamin B12 (cobalamin) is essential for DNA synthesis, hematopoiesis, and neurological function [1]. While subclinical cobalamin deficiency is frequently observed in the general population with a prevalence of up to 26%, clinical B12 deficiency with hematological or neurological manifestations is uncommon [2].

Among the clinical manifestations of cobalamin deficiency are reversible megaloblastic anemia and neurologic and neuropsychiatric disorders such as dorsal column dysfunction, peripheral neuropathy, delirium, psychosis, and dementia [1]. In severe cases, B12 deficiency may manifest as pseudo-thrombotic microangiopathy (pseudo-TMA), which is characterized by microangiopathic hemolytic anemia with schistocytosis, low haptoglobin, high lactate dehydrogenase, thrombocytopenia, and reticulocytopenia [3]. This is a rare presentation that only occurs in around 2.5% of patients with cobalamin deficiency and is often misdiagnosed as thrombotic thrombocytopenic purpura (TTP), resulting in misguided treatment [4].

In adults, the major risk factor for developing B12 deficiency is pernicious anemia, an autoimmune disease caused by autoantibodies against intrinsic factor or gastric parietal cell antigens, which prevents the vitamin B12-intrinsic factor complex, leading to a decrease in vitamin B12 absorption in the terminal ileum [2]. Other etiologies include insufficient dietary intake, malabsorption disorders, and genetic conditions [4].

We present the case of a patient with pseudo-TMA caused by B12 deficiency secondary to undiagnosed pernicious anemia.

## Case presentation

An 89-year-old female with a known past medical history including essential hypertension and dyslipidemia presented to the Emergency Department with nausea, vomiting, and abdominal pain persisting for two days. She reported that the abdominal pain was diffuse and non-radiating. The patient also complained of two weeks of weakness, fatigue, and dyspnea on exertion. She denied fever, diarrhea, weight loss, blood loss, numbness or tingling, tobacco, illicit drugs, or alcohol consumption, as well as newly introduced medication or herbal supplements. There was no known personal or family history of malignancy or hematologic or autoimmune disorders.

Upon admission, her vital signs were unremarkable. Physical evaluation revealed conjunctival pallor, diffuse abdominal pain without hepatosplenomegaly, and crackles at the bases evidenced on pulmonary auscultation. Cardiac auscultation was normal, and there was no lower limb edema. A skin examination showed no petechiae, ecchymoses, or hematomas. Neurological examination was normal with intact sensation and motor functions bilaterally.

Laboratory testing revealed pancytopenia and macrocytosis with hemoglobin at 5.6g/dL (reference range [RR]: 12.0-15.0g/dL), leukocytes at 2.9×10^3^/μL (RR: 4.0-10.0×10^3^/μL), platelet count of 120,000/μL (RR: 150,000-400,000/μL), hematocrit of 15.6% (RR: 36.0-46.0%), mean corpuscular volume (MCV) of 120fL (RR: 80-97fL), and reticulocytes at 3.6% (RR: 0.2-2.0%). Unconjugated hyperbilirubinemia was noted with a total bilirubin of 3.0mg/dL (RR: <1.0mg/dL) and direct bilirubin of 1.2mg/dL (RR: 0-0.3mg/dL). Lactate dehydrogenase (LDH) was elevated at 818IU/L (RR: 81-234IU/L) and haptoglobin was low at <0.06 mg/dL (RR: 0.3-2.0mg/dL). Laboratory findings also showed acute kidney failure with urea at 81mg/dL (RR: <50mg/dL) and creatinine at 1.83mg/dL (RR: 0.55-1.02mg/dL), as well as an elevated pro-BNP (brain natriuretic peptide) of 33,268pg/mL (RR: <1,800pg/mL). The peripheral blood smear showed hypersegmented neutrophils, dacrocytes, severe anisopoikilocytosis, and rare schistocytes.

Deeming the hypothesis of a thrombotic microangiopathy (TMA), the PLASMIC score [5] was calculated, which rendered five points, which provided an intermediate probability of TTP, and warranted plasmapheresis’ consideration.

Further laboratory workup was significant for severe vitamin B12 deficiency (96pg/mL, RR: 211-911pg/mL), folate deficiency (4.6ng/mL, RR: >5.38ng/mL), negative direct antiglobulin and antinuclear antibody tests, and normal iron and coagulation studies. The reticulocyte production index (RPI) was low at 0.53, suggesting a hypoproliferative bone marrow, which is atypical for hemolytic anemia that commonly presents as hyperproliferative. Additionally, autoantibodies to intrinsic factor and parietal cells were positive, consistent with pernicious anemia.

The relevant laboratory findings are presented in Table 1.

**Table 1:** Laboratory findings BNP, brain natriuretic peptide; Cr, creatinine; DBil, direct bilirubin; Hgb, hemoglobin; Ht, hematocrit; LDH, lactate dehydrogenase; MCV, mean corpuscular volume; TBil, total bilirubin

	Laboratory value	Reference range
Hgb (g/dL)	5.6	12.0–15.0
Ht (%)	15.6	36.0–46.0
MCV (fL)	120	80–97
Leukocytes (x10³/µL)	2.9	4.0–10.0
Platelets (µL)	120,000	150,000–400,000
Reticulocytes (%)	3.6	0.2–2.0
TBil (mg/dL)	3.0	<1.0
DBil (mg/dL)	1.2	0–0.3
LDH (IU/L)	818	81–234
Haptoglobin (mg/dL)	<0,06	0.3–2.0
Urea (mg/dL)	81	<50
Cr (mg/dL)	1.83	0.55–1.02
Pro-BNP (pg/mL)	33268	<1800
Vitamin B12 (pg/mL)	96	211–911
Folate (ng/mL)	4.6	>5.38

**Figure 1:**
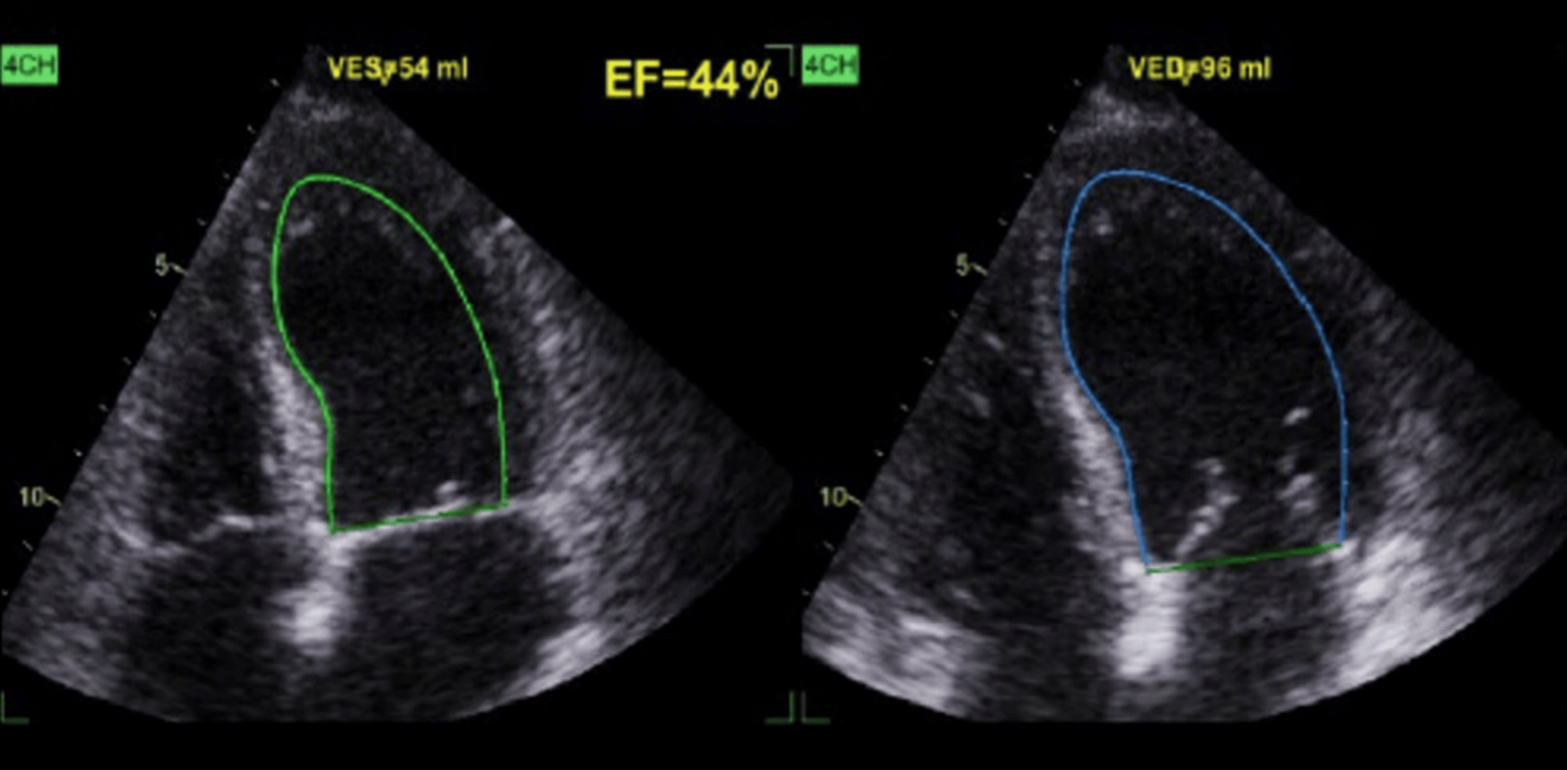
Thoracic echocardiography (four-chamber view) of the patient. The patient's thoracic echocardiography reveals a mildly reduced ejection fraction without any structural cardiac abnormalities. The image highlights the EF measurement. EF, ejection fraction; VED, volume end-diastole; VES, volume end-systole

Computed tomography scan of the thorax, abdomen, and pelvis showed bilateral pleural effusion but did not depict hepatosplenomegaly or neoplastic disease. A thoracic echocardiography revealed a mildly reduced ejection fraction without any structural cardiac abnormalities (Figure 1). A myelogram and bone marrow biopsy were performed, which were suggestive of megaloblastic anemia.

The final diagnosis was pseudo-TMA due to B12 deficiency secondary to pernicious anemia. She was transfused with two units of packed red blood cells and was started on daily intramuscular cobalamin injections of 1,000μg. Folic acid supplementation, diuretic therapy, and heart failure prognosis-modifying agents were also instituted. She reported resolution of her symptoms, and the blood counts began to improve across all cell lineages.

The patient was discharged with follow-up in an Internal Medicine consultation with weekly cobalamin injections for four weeks and monthly afterward.

## Discussion

Cobalamin is a vitamin commonly found in meat, fish, and dairy products that is absorbed in the terminal ileum after binding to the intrinsic factor produced by gastric parietal cells [3]. It plays a physiological role in DNA synthesis, hematopoiesis, and neurological function [1]. Vitamin B12 deficiency can result in ineffective intramedullary hematopoiesis and demyelination; however, deficiency leading to hemolysis is uncommon, seen in approximately 10% of the cases, and, among these, only up to 2.5% present with pseudo-TMA [6].

TMA is a term used to describe a heterogeneous group of diseases that is characterized by microvascular endothelial injury and thrombosis [7]. Its classification is based on the etiopathogenesis of the diseases, with hemolytic uremic syndrome and TTP being the two main variants of TMA [8]. Beyond these entities, several other disorders may be associated with TMA and are generally referred to as secondary TMA. These etiologies include severe hypertension, systemic infections (such as invasive pneumococcal infections), autoimmune diseases, drug-induced TMA, disseminated malignancies, inborn errors of cobalamin metabolism, and TMA related to transplantation or pregnancy [7,8].

Given the rapid and severe progression of TMA, identifying plausible etiologies and precipitating factors is important to ensure supportive and/or targeted therapies, which may vary significantly [8]. TMAs should be considered in patients presenting with the triad of a Coombs-negative microangiopathic hemolytic anemia (MAHA), consumptive thrombocytopenia, and platelet-mediated microvascular occlusion, leading to organ failure [6]. MAHA is characterized by low hemoglobin, schistocytes in peripheral blood, elevated lactate dehydrogenase, low haptoglobin, and increased indirect bilirubin. Normal coagulation tests help distinguish TMA from disseminated intravascular coagulation [7,8].

In patients presenting with these clinical findings, it is paramount to distinguish pseudo-TMA from TMA, particularly TTP, as it is the most comparable condition and the primary TMA to consider in adult patients. TTP is caused by reduced activity of the ADAMTS-13, a von Willebrand factor-cleaving protease, which leads to intravascular platelet aggregates or microthrombi with consequent end-organ ischemic damage [3]. In contrast, vitamin B12 deficiency is believed to cause intramedullary hemolysis, leading to peripheral cytopenias, and homocysteine accumulation. These mechanisms are thought to result in fragile erythrocytes and their destruction, producing schistocytosis [3,4,6].

While TTP is a life-threatening disorder that warrants prompt plasma exchange, as the mortality is around 80-90% if left untreated, pseudo-TMA due to vitamin B12 deficiency is unresponsive to plasmapheresis and is treated with cobalamin supplementation [3]. The rarity of cobalamin deficiency-related pseudo-TMA makes its diagnosis challenging, with 38.8% of those patients being initially misdiagnosed with TTP and treated with plasma product therapy, including plasmapheresis [9].

While some features of pseudo-TMA are similar to those of TTP, there are some significant differences. Reticulocytopenia in cobalamin deficiency-related pseudo-TMA was recognized as a universal finding in helping differentiate it from other causes of hemolysis [1]. Hence, RPI helps in differentiating between TTP and pseudo-TMA; while in the former, the RPI is greater than 3, in the latter, it is commonly less than 2 [3]. In contrast to TTP, in which there is no lack of substrate for DNA synthesis and a compensatory increase in erythropoiesis is observed, in vitamin B12 deficiency, there is a suppression in the bone marrow’s production of hematopoietic cells, leading to ineffective erythropoiesis, as reflected by a low reticulocyte count [3,10].

Furthermore, while high levels of LDH are common in pseudo-TMA as it is released during the intramedullary hemolysis of immature nucleated erythrocyte precursors, that is not expected in TTP since the mature erythrocytes being lysed in the peripheral vasculature are anucleate [3,4,6,11]. Moreover, it is suggested that thrombocytopenia associated with TTP is generally severe, while in pseudo-TMA it is mild to moderate [1,10,11], and that there is only modest unconjugated hyperbilirubinemia in pseudo-TMA compared to TTP, as erythroid progenitors contain less hemoglobin [3,4,10]. Additionally, the presence of macrocytosis is another indicator of pseudo-TMA, but schistocytosis may falsely lower the MCV [6], and the detection of hypersegmented neutrophils in the peripheral blood smear should prompt suspicion of cobalamin deficiency [11].

In patients with a TMA-like clinical presentation, the presence of risk factors for cobalamin deficiency should raise suspicion of pseudo-TMA, and these include poor nutrition, alcoholism, strict vegetarian or vegan diets, autoimmune diseases, or gastric surgery [11].

The PLASMIC score is a clinical scoring system that estimates the probability of TTP that allows the initiation of life-saving treatment while awaiting ADAMTS-13 levels, but it cannot be reliably used to exclude pseudo-TMA due to the overlapping features with TTP [11].

## Conclusions

Vitamin B12 deficiency can present with a wide spectrum of manifestations, including pseudo-TMA. This rare presentation is frequently misdiagnosed as primary TMA syndromes, particularly TTP, due to shared features such as microangiopathic hemolytic anemia, thrombocytopenia, and organ failure. Although there are no established diagnostic criteria for this condition, several distinguishing features can support the diagnosis of pseudo-TMA, including a low RPI.

Due to the overlap in clinical presentation with TTP, patients with pseudo-TMA risk receiving potentially harmful treatments, such as plasma exchange, to which they are unresponsive, potentially delaying appropriate therapy with vitamin B12 supplementation.
